# Variation in competence for ZIKV transmission by *Aedes aegypti* and *Aedes albopictus* in Mexico

**DOI:** 10.1371/journal.pntd.0006599

**Published:** 2018-07-02

**Authors:** Selene M. Garcia-Luna, James Weger-Lucarelli, Claudia Rückert, Reyes A. Murrieta, Michael C. Young, Alex D. Byas, Joseph R. Fauver, Rushika Perera, Adriana E. Flores-Suarez, Gustavo Ponce-Garcia, Americo D. Rodriguez, Gregory D. Ebel, William C. Black

**Affiliations:** 1 Department of Microbiology, Immunology and Pathology, Colorado State University, Fort Collins, Colorado, United States of America; 2 Universidad Autonoma de Nuevo Leon, Facultad de Ciencias Biologicas, San Nicolas de los Garza, Nuevo Leon, México; 3 Centro Regional de Investigación en Salud Publica, Instituto Nacional de Salud Publica, Tapachula, Chiapas, México; The Connecticut Agricultural Experiment Station, UNITED STATES

## Abstract

**Background:**

ZIKV is a new addition to the arboviruses circulating in the New World, with more than 1 million cases since its introduction in 2015. A growing number of studies have reported vector competence (VC) of *Aedes* mosquitoes from several areas of the world for ZIKV transmission. Some studies have used New World mosquitoes from disparate regions and concluded that these have a variable but relatively low competence for the Asian lineage of ZIKV.

**Methodology/Principal findings:**

Ten *Aedes aegypti* (L) and three *Ae*. *albopictus* (Skuse) collections made in 2016 from throughout Mexico were analyzed for ZIKV (PRVABC59—Asian lineage) VC. Mexican *Ae*. *aegypti* had high rates of midgut infection (MIR), dissemination (DIR) and salivary gland infection (SGIR) but low to moderate transmission rates (TR). It is unclear whether this low TR was due to heritable salivary gland escape barriers or to underestimating the amount of virus in saliva due to the loss of virus during filtering and random losses on surfaces when working with small volumes. VC varied among collections, geographic regions and whether the collection was made north or south of the Neovolcanic axis (NVA). The four rates were consistently lower in northeastern Mexico, highest in collections along the Pacific coast and intermediate in the Yucatan. All rates were lowest north of the NVA. It was difficult to assess VC in *Ae*. *albopictus* because rates varied depending upon the number of generations in the laboratory.

**Conclusions/Significance:**

Mexican *Ae*. *aegypti* and *Ae*. *albopictus* are competent vectors of ZIKV. There is however large variance in vector competence among geographic sites and regions. At 14 days post infection, TR varied from 8–51% in *Ae*. *aegypti* and from 2–26% in *Ae*. *albopictus*.

## Author summary

*Aedes aegypti* is an efficient vector for arboviruses which is partially determined by its vector competence (VC) status, a highly variable trait. Based on previous reports, the VC of New World mosquitoes is limited for ZIKV. However, a VC assessment from additional geographical sources was lacking. Therefore, we evaluated the VC for ZIKV using recently colonized mosquitoes from Mexico. *Aedes aegypti* and *Ae*. *albopictus* were highly susceptible to ZIKV infection but varied greatly in their transmission rates. As with previous studies of other flaviviruses; VC for ZIKV was highly variable in both *Ae*. *aegypti* and *Ae*. *albopictus* but was greatest in *Ae*. *aegypti*, supporting its role as the main ZIKV vector.

## Introduction

Zika virus (ZIKV, Flavivirus, Flaviviridae) was first isolated from a febrile sentinel rhesus macaque in the Zika forest of Uganda in 1947 and later in 1948 from *Ae*. *africanus* mosquitoes from the same area [1]. ZIKV circulated in Africa and Asia without much attention until 2007 when a major outbreak occurred in the Pacific Island of Yap in the Federate States of Micronesia [2, 3]. Outbreaks were later reported in other Pacific islands: French Polynesia, Easter Island, the Cook Islands and New Caledonia during 2013–2014 [4–6]. Making its arrival to the Americas in early 2015, ZIKV circulation was confirmed in Brazil in May and, as expected, ZIKV spread quickly to areas where the vectors were present. Mosquito-borne transmission has been reported in 48 countries of the Americas since its introduction [7]. In addition, ZIKV was associated with congenital abnormalities such as microcephaly and an increased incidence of Guillain-Barré syndrome, and was thus declared a Public Health Emergency of International Concern by the World Health Organization on February 1, 2016 [8], which ended nine months later [9]. Since its introduction, the Pan American Health Organization has reported more than a thousand cumulative Zika cases in the Americas. Mexico alone had a total of 129 cases [10], with its first case of congenital ZIKV syndrome in November of 2016 [11].

The main mechanism of ZIKV transmission in epidemic and endemic areas is through the bite of an infectious mosquito, with *Ae*. *aegypti* apparently serving as the primary vector [12]. From the screening of wild-caught mosquitoes in Mexico, ZIKV RNA has been detected in *Ae*. *aegypti* pools collected in and around houses of suspected ZIKV cases [13]. *Aedes albopictus* [14] have also been confirmed to be infected with ZIKV.

Vectorial capacity is a quantitative measure of the potential of an arthropod vector to transmit a pathogen. It is defined as the average number of potentially infective bites that will ultimately be delivered by all the vectors feeding on a single host in a day [15]. Vectorial capacity is impacted by extrinsic factors like vector density, vector longevity, length of the extrinsic incubation period (EIP) and blood feeding behavior [16, 17] and also by intrinsic factors like vector competence (VC). VC is defined as the intrinsic ability of an arthropod vector to acquire, maintain and eventually transmit a pathogen [18]. Upon ingestion, the arbovirus has to replicate to be transmitted to a susceptible host in a subsequent feeding episode. However the virus has to first bypass a series of physiological and anatomical barriers [19]. Briefly, upon entry of the virus into the mosquito midgut through an infectious blood meal, the virus has to establish an infection; if this does not occur the mosquito has a midgut infection barrier (MIB). Next, the virus has to replicate in the midgut and then escape the midgut to disseminate to other tissues. When this does not occur the mosquito is said to have a midgut escape or dissemination barrier (MEB). The virus may infect several mosquito tissues including, most importantly, the salivary glands where it again has to establish an infection. If this is prevented the mosquito has a salivary gland infection barrier (SGIB). Finally, the virus has to replicate and disseminate into the saliva from where it will be expectorated with the saliva while probing and feeding in a susceptible vertebrate host. If this is limited, the mosquito has a salivary gland escape barrier (SGEB) [19, 20]. Consecutively the MIB, MEB, SGIB and SGEB contribute to the overall VC phenotype.

By harvesting mosquitoes at 7 and 14 days post infection (dpi) we can obtain potential indicators of infection and dissemination and/ or transmission respectively [21, 22]. Previous studies have reported low ZIKV transmission rates for the Asian lineage of ZIKV using mosquitoes from a wide geographical range in the New World [23, 24]. We hypothesized that VC is variable and is highly dependent upon the geographic origin of the mosquito populations. Hence, we analyzed the ZIKV transmission potential of 13 recently colonized *Aedes* collections, 10 of *Ae*. *aegypti* and 3 of *Ae*. *albopictus*, from different locations across Mexico. These collections were analyzed for ZIKV (strain PRVABC59—Asian genotype) VC at 7 and 14 days dpi.

Herein we report that both *Aedes* species are competent for ZIKV transmission and that MIB, MEB, SGIB and SGEB vary by species, as well as by collection, region, and whether they were collected north or south of the NVA. TR ranged from 2–51% at 7 dpi and from 8–51% at 14 dpi in *Ae*. *aegypti*. *Aedes albopictus* had from 0–8% transmission at 7 dpi and 2–26% at 14 dpi. We describe the contribution of each of the barriers for ZIKV transmission showing that a SGEB may be an important barrier to ZIKV transmission in *Ae*. *aegypti* populations.

## Methods

### Mosquitoes

Collection protocols were approved by the ethics board at the Universidad Autonoma de Nuevo Leon. Written informed consents were obtained from the household owners for mosquito collections indoor and outside the houses. No special permit was needed for sampling in non-private properties.

**Fig 1:**
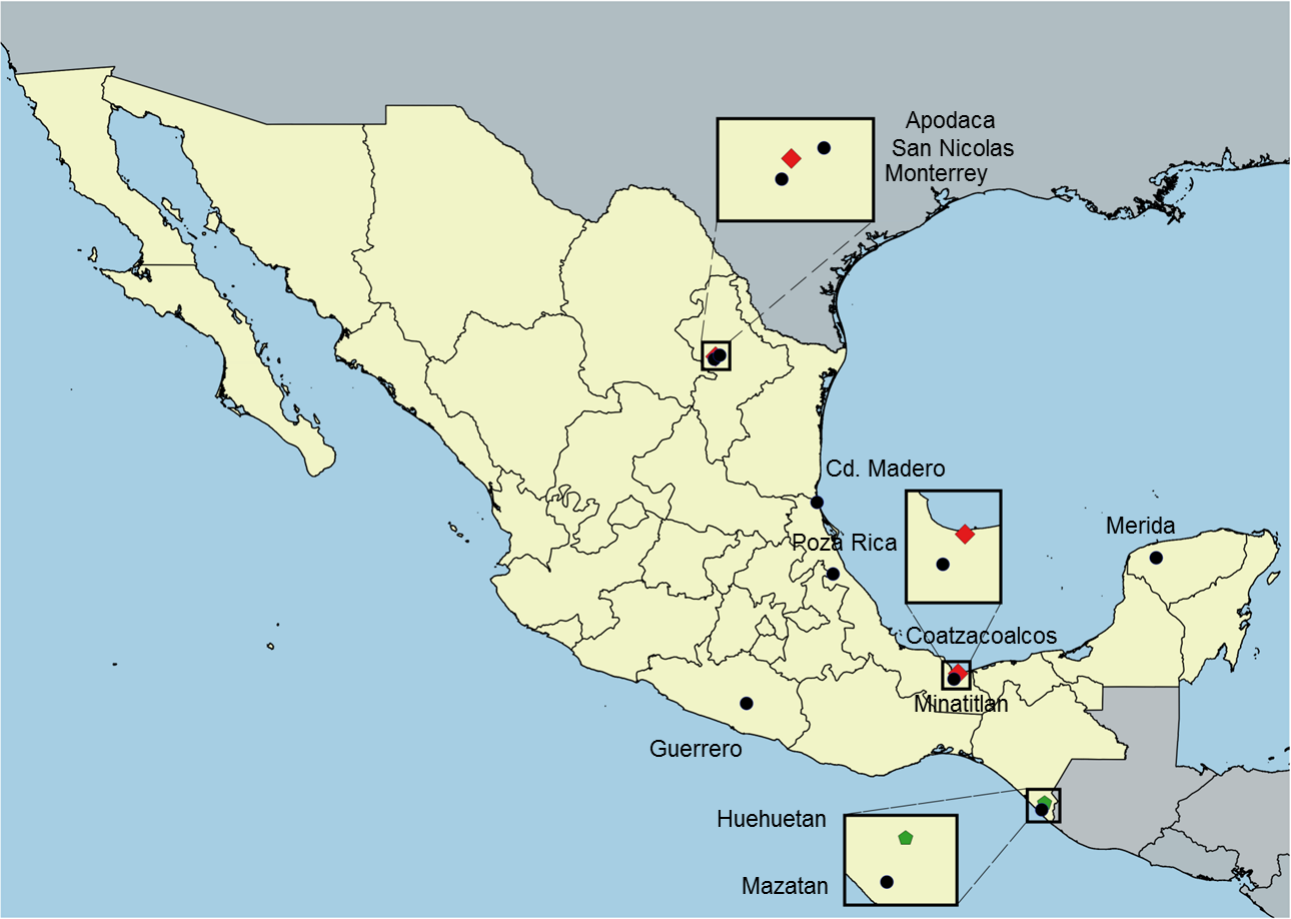
Map showing the collection sites of *Aedes* mosquitoes. Red diamonds indicate where both species were collected. Black dots depict *Ae*. *aegypti* collections and green dots indicate *Ae*. *albopictus* collections. Free access QGIS (2.8.1) Wien software with public access layers (USA_adm0, NIC_adm0, HND_adm0, GTM_adm0, MEX_adm1) was used for map elaboration.

*Aedes* eggs were collected from ovitraps set at different locations in Mexico (Fig 1) during 2016 with exception of the collections from the state of Chiapas (Huehuetan and Mazatan) where immature stages were obtained from at least 20 different containers (S1 Table). All 10 *Ae*. *aegypti* collections were analysed from north to south and were further grouped into regions (Northeastern, Yucatan, or Pacific). These regions are defined based upon a past survey of variation in mitochondrial DNA in 38 collections across Mexico [25]. That study indicated that northeastern Mexico collections were genetically differentiated from and had lower genetic diversity than Yucatan and Pacific coastal collections. Regions were further grouped as to whether they were located north or south of the NVA based upon earlier findings [26].

At each location where ovitraps were used, 4–5 were set and checked once a week. The eggs were dried and shipped to the laboratory at Colorado State University (PHS permit no. 2016-06-185), where they were hatched, reared to adults and then identified to species. Larvae were fed *ad libitum* with a 10% (w/v) liver powder solution. Adult mosquitoes were maintained on sucrose *ad libitum* and for egg production citrated sheep blood was given once a week through water-jacketed glass feeders using hog gut as a membrane through which to feed. Adults were maintained at insectary conditions (28°C, 70% relative humidity and 12:12 light:dark diurnal cycle). Mosquitoes were identified as *Ae*. *aegypti* or *Ae*. *albopictus* based on scale patterns on the thorax after adult eclosion [27].

### Mosquito infections

**Fig 2:**
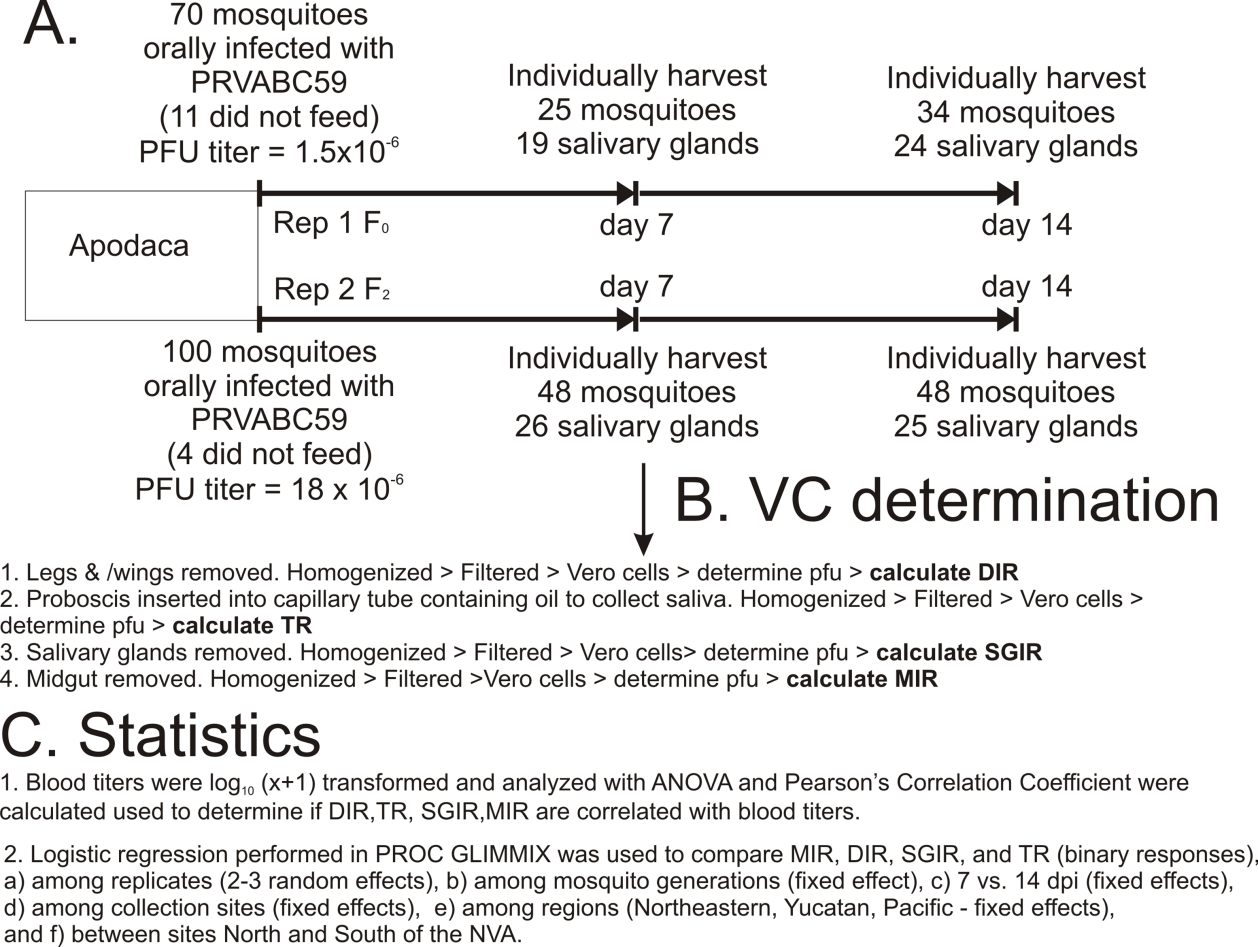
Flow chart indicating. A) how mosquitoes from the Apodaca collection were processed at 7 dpi and 14 dpi. B) how each mosquito in the Apodaca collection was processed to determine DIR, TR, SGIR, MIR. C) and how each collection was processed statistically.

The flow chart in Fig 2 indicates how each of the 13 collections were evaluated for VC using *Ae*. *aegypti* collected in Apodaca as an example (raw data in first four rows of S1 Table). The ZIKV strain used for these studies was PRVABC59 (Accession # KU501215) [28] obtained from the CDC. PRVABC59 strain had been passed four times on African green monkey kidney cells (Vero, ATCC CCL-81). For mosquito infections PRVABC59 was used to infect Vero cells at a MOI of 0.01. After 4 days infection, the supernatant was harvested and centrifuged at 3,000xg for 10 min at 4°C. The supernatant was then transferred to a clean tube and a sample was taken to perform ZIKV quantification by quantitative-reverse transcriptase PCR (RT-qPCR) with oligonucleotides for the ZIKV 3’ untranslated region (S2 Table) prior to the infection of mosquitoes. During this time, the supernatant was maintained at 4°C until it was mixed with blood. RNA was extracted from 50 μL of the clarified supernatant using the Direct-zol™ RNA MiniPrep Kit (Zymo Research Corp.) following manufacturer recommendations. Based upon the result, the supernatant was supplemented with Dulbecco’s modified Eagle’s medium (DMEM) and 20% FBS and further mixed 1:1 with defibrinated calf blood to a final concentration of 1 x10^9^ genome equivalents (GE) / mL. Viral titers in the ZIKV infectious calf blood were confirmed by plaque assays on Vero cells, averaging 10^6^ PFU/mL (Fig 2A).

Prior to feeding, 5–6 day old mosquitoes were deprived of sucrose and water for 24 hours. Mosquito infections were performed under BSL-3 containment where they were offered a ZIKV infectious blood meal through water-jacketed glass feeders covered with hog gut. After up to one-hour of feeding, mosquitoes were cold-anesthetized and engorged females were placed into new cartons and water and a sugar source were provided (Fig 2A.)

### Vector competence assessment

At 7 and 14 dpi, mosquitoes were cold anesthetized at 4°C. To measure the DIR, mosquito legs and wings were removed and placed into a tube with 250 μL mosquito diluent (1X phosphate buffer saline (PBS) supplemented with 20% heat-inactivated fetal bovine serum (FBS), 50 μg/mL penicillin/streptomycin, 50 μg/mL gentamycin, 2.5 μg/mL fungizone) (Fig 2B) and a stainless steel bead for homogenization. To obtain saliva to measure TR, the mosquito (without legs or wings) proboscis was placed into a capillary tube that contained immersion oil (~5 μL) and allowed to expectorate saliva for 30 minutes. Following salivation, the tip of the capillary tube was broken into a tube containing 100 μL of mosquito diluent. Subsequently, the midgut (to measure MIR) and salivary glands (to measure SGIR) were dissected, rinsed individually in PBS and placed in tubes with mosquito diluent and a stainless steel bead. Forceps were dipped in 70% ethanol and cleaned after each tissue was dissected and between individual mosquitoes. Mosquito tissues were stored at -80°C until further processing (Fig 2B).

Mosquito tissues (midguts, legs/wings and salivary glands) were thawed and homogenized at 25 cycles/second for one minute using a Retsch Mixer Mill MM400 (Germany) and centrifuged at 20,000xg for 5 minutes at 4°C while saliva samples were centrifuged at 20,000 x g for 3 minutes at 4°C, mixed by vortexing and centrifuged for 3 additional minutes. Clarified supernatant was titrated by plaque assay on Vero cells to determine whether individual mosquito tissues contained infectious ZIKV (Fig 2B).

### Plaque assays

Plaque assays were performed on Vero cells which were maintained in DMEM containing 8% FBS, 50 μg/mL penicillin and streptomycin and 50 μg/mL gentamycin at 37°C with 5% CO_2_. Twelve-well plates were seeded with Vero cells and allowed to reach 90 to 95% confluency. Media was then removed and replaced with 250 μL of DMEM containing 1% FBS, 50 μg/mL penicillin and streptomycin, and 50 μg/mL gentamycin. Subsequently, each sample (30 μL for mosquito saliva or 70 μL for midgut, salivary glands and legs/wings) was added to one well of the plate. The plates were rocked for 90 minutes to allow absorption after which 1 mL of overlay (tragacanth gum (6 g/L) in 1X DMEM supplemented with 10% FBS, 50 μg/mL penicillin/ streptomycin and 50 μg/mL gentamycin) was added to each well and plates were incubated at 37°C with 5%CO_2_. After 5 days, the plates were fixed with a staining solution (1 g/L crystal violet in 20% ethanol solution); plaques were visualized on a light box and recorded as plaque positive or negative. We used 10 fold dilutions for virus (blood meal) titration. But for testing the presence of the virus in the mosquito tissues or saliva samples we determined the presence of ZIKV infectious particles by observing plaques from a fixed volume of the sample (Fig 2B).

### Data and statistical analysis

We determined the rates of midgut infection, dissemination, salivary gland infection and transmission in each of the mosquito populations tested [29, 30]. The dissemination rate (DIR) was defined as the number of mosquitoes with infectious ZIKV in the legs/wings divided by the number of blood-fed mosquitoes (Fig 2B.1). The transmission rate (TR) was defined as the number of mosquitoes whose saliva contained infectious ZIKV divided by the total number of salivary glands that were successfully dissected (Fig 2B.2). Salivary gland infection rate (SGIR) was defined as the number of mosquitoes with infectious ZIKV in the salivary glands divided by number of salivary glands that were successfully dissected (Fig 2B.3). Midgut infection rate (MIR) was defined as the number of mosquitoes with infectious ZIKV in the midgut divided by the total number of mosquitoes that had blood-fed. (Fig 2B.4).

Next we calculated the additive contribution of each of the four transmission barriers to blocking transmission by adjusting MIR, DIR, SGIR and TR so that they sum to 100%.

AMIR=numberwithinfectedmidgut/numberbloodfed (1)

ADIR=numberwithinfectedlegs/numberwithinfectedmidgut (2)

ASGIR=numberwithinfectedsalivarygland/numberwithinfectedlegs (3)

ATR=numberwithinfectedsaliva/numberwithinfectedsalivarygland (4)

VC=numberwithinfectedsaliva/numberbloodfed (5)

VC=AMIRxADIRxASGIRxATR (6)

$$Log_{10}\;\left(VC\right)=Log_{10}\;\left(AMIR\right)+Log_{10}\;\left(ADIR\right)+Log_{10}\;\left(ASGIR\right)+Log_{10}\;(ATR)$$ (7)

$$\%MIB=Log_{10}\;\left(AMIR\right)/\;Log_{10}\;\left(VC\right)\;x\;100$$ (8)

$$\%MEB=Log_{10}\;\left(ADIR\right)/\;Log_{10}\;\left(VC\right)\;x\;100$$ (9)

$$\%SGIB=Log_{10}\;\left(ASGIR\right)/\;Log_{10}\;\left(VC\right)\;x\;100$$ (10)

$$\%SGEB=Log_{10}\;\left(ATR\right)/\;Log_{10}\;\left(VC\right)\;x\;100$$ (11)

GraphPad PRISM version 7.03 (GraphPad Software, San Diego, CA, USA) and SigmaPlot 12 (Systat Software, Inc, San Jose, CA) were used for graph construction.

Blood titers were log_10_ (x+1) transformed (Fig 2C.1). All analyses were replicated at least twice with different (independent) virus preparations for each replicate (S1 Table). MIR, DIR, SGIR, and TR were compared using logistic regression in PROC GLIMMIX in SAS 9.4. The LSMEANS option was used to report mean proportions and their 95% confidence intervals. Main effects in the logistic regression were mosquito generation, dpi, collection, geographic region and whether they were from north or south of the NVA [26]. Blood meal titers (pfu/mL) were compared using Analysis of Variance (ANOVA) in PROC GLM in SAS 9.4 (Fig 2C.3). Pearson’s Correlation Coefficients were calculated using PROC CORR (Fig 2C.3). Proportions (MIR, DIR, SGIR, TR) was normalized with arcsine of the square root prior to ANOVA and correlation analyses.

## Results

### Effects of mosquito generations and virus preparations on VC

Due to time and space constraints it was not feasible to measure the four components of vector competence (MIR, DIR, SGIR, TR) simultaneously in all 13 *Aedes* collections. Instead 6 generations (F_1_—F_6_) (S1 Table) of *Ae*. *aegypti* and the three generations of *Ae*. *albopictus* were required to complete all experiments. The PRVABC59 virus was grown fresh for each round of infections and titers varied significantly from 5.1 to 7.6 logs among the seven generations of *Ae*. *aegypti* (ANOVA P = 0.0252) and the three generations of *Ae*. *albopictus* (ANOVA P = 0.0203). In *Ae*. *aegypti* the blood titer was not correlated with 7 dpi MIR (r = 0.246, P = 0.2832), DIR (r = 0.046, P = 0.8420), SGIR (r = 0.157, P = 0.5210) or TR (r = 0.147, P = 0.525). The same was the case for 14 dpi MIR (r = 0.350, P = 0.1103), DIR (r = 0.314, P = 0.1541), SGIR (r = 0.304, P = 0.1800) or TR (r = 0.415, P = 0.0546). All correlation analyses had n = 22 observations. Even though infectious blood titers varied among the different mosquito generations they did not appear to affect vector competence parameters in *Ae*. *aegypti*.

Interestingly these same patterns did not hold for the three *Ae*. *albopictus* collections. S1–S4 Figs show that the four VC parameters vary greatly among the three generations. Furthermore, the four VC parameters were correlated with 7dpi MIR (r = 0.693, P = 0.127), DIR (r = 0.905, P = 0.0132), SGIR (r = 0.960, P = 0.0024) and Sal (r = 0.976, P = 0.0009) and at 14 dpi MIR (r = 0.765, P = 0.0452), DIR (r = 0.830, P = 0.0208), SGIR (r = 0.886, P = 0.0079) but not TR (r = 0.545, P = 0.2064). All correlation analyses had n = 7 observations. Both trends suggest that all four VC parameters are confounded by the large variation among the generations of *Ae*. *albopictus* measured. For these reasons, results can be seen in S1–S4 Figs but are not considered further.

### MIR, DIR, SGIR, and TR among *Ae*. *aegypti* generations, dpi, collections and regions

**Fig 3:**
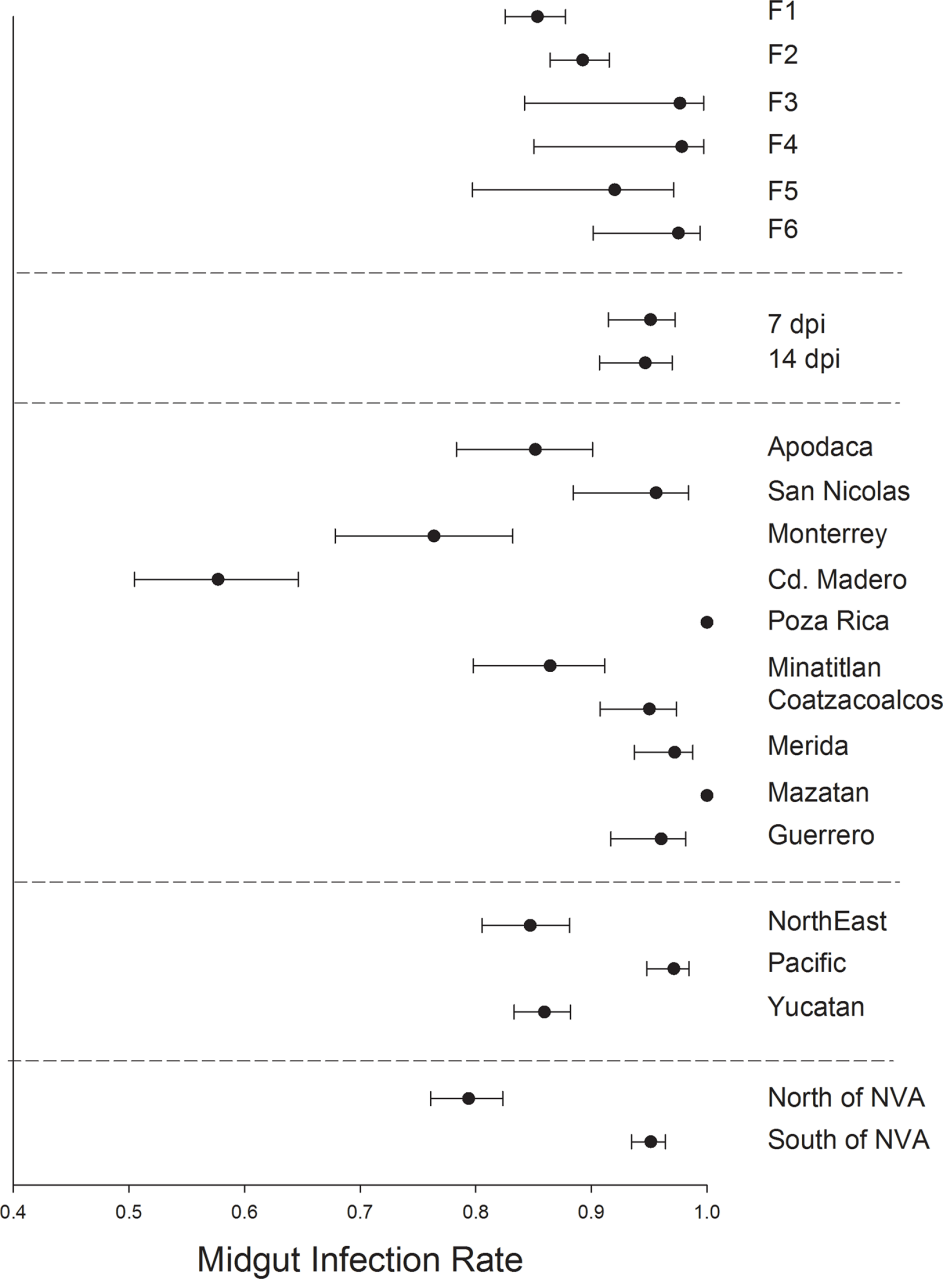
Logistic regression and least square means to compare midgut infection rates. 1) among six generations of *Ae*. *aegypti*, 2) between mosquitoes processed at 7 versus 14 dpi, 3) among 10 collections, 4) among 3 regions and 5) between collections north versus south of the NVA.

In *Ae*. *aegypti* MIR varied significantly between mosquito generations F_1_ and F_6_ but otherwise broadly overlapped (Fig 3). MIR was similar between mosquitoes harvested at 7 or 14 dpi. MIR varied broadly between collections with Ciudad Madero and Monterrey having the lowest MIR and Poza Rica and Mazatan having the highest MIR. MIR was least in northeastern and Yucatan regions and MIR was highest in the Pacific collections. MIR was lowest in collections south of the NVA.

**Fig 4:**
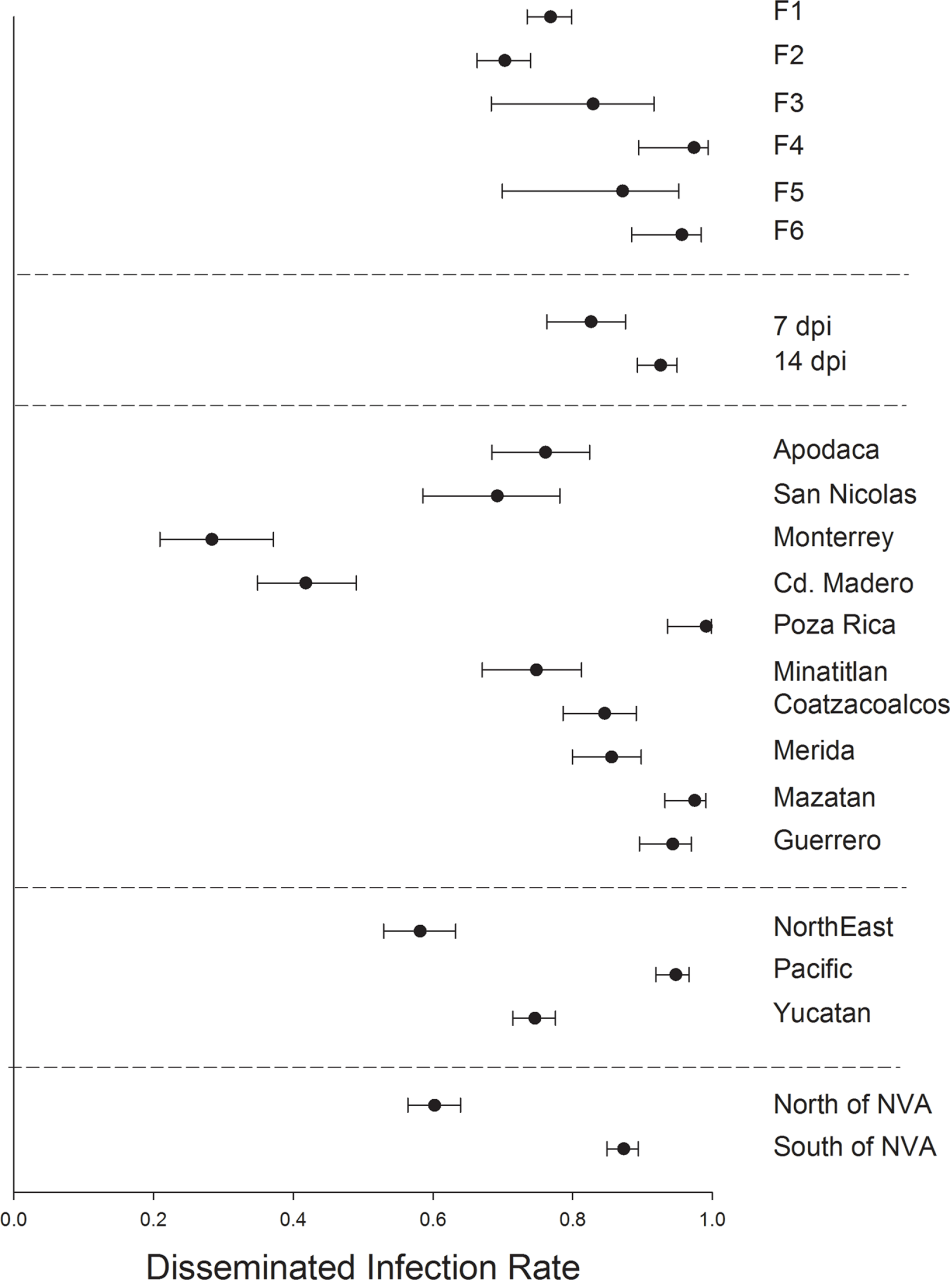
Logistic regression and least square means to compare disseminated infection rates. 1) among six generations of *Ae*. *aegypti*, 2) between mosquitoes processed at 7 versus 14 dpi, 3) among 10 collections, 4) among 3 regions and 5) between collections north versus south of the NVA.

DIR varied significantly between mosquito generations F_1_, F_2_ and F_6_ but otherwise broadly overlapped among the six mosquito generations (Fig 4). Mosquitoes harvested at 7 dpi had a lower DIR than those harvested at 14 dpi. DIR varied broadly between collections with Ciudad Madero and Monterrey having the lowest DIR and Poza Rica and Mazatan having the greatest DIR. DIR was least in northeastern collections, intermediate in the Yucatan and highest in the Pacific collections. DIR was lowest in collections north of the NVA.

**Fig 5:**
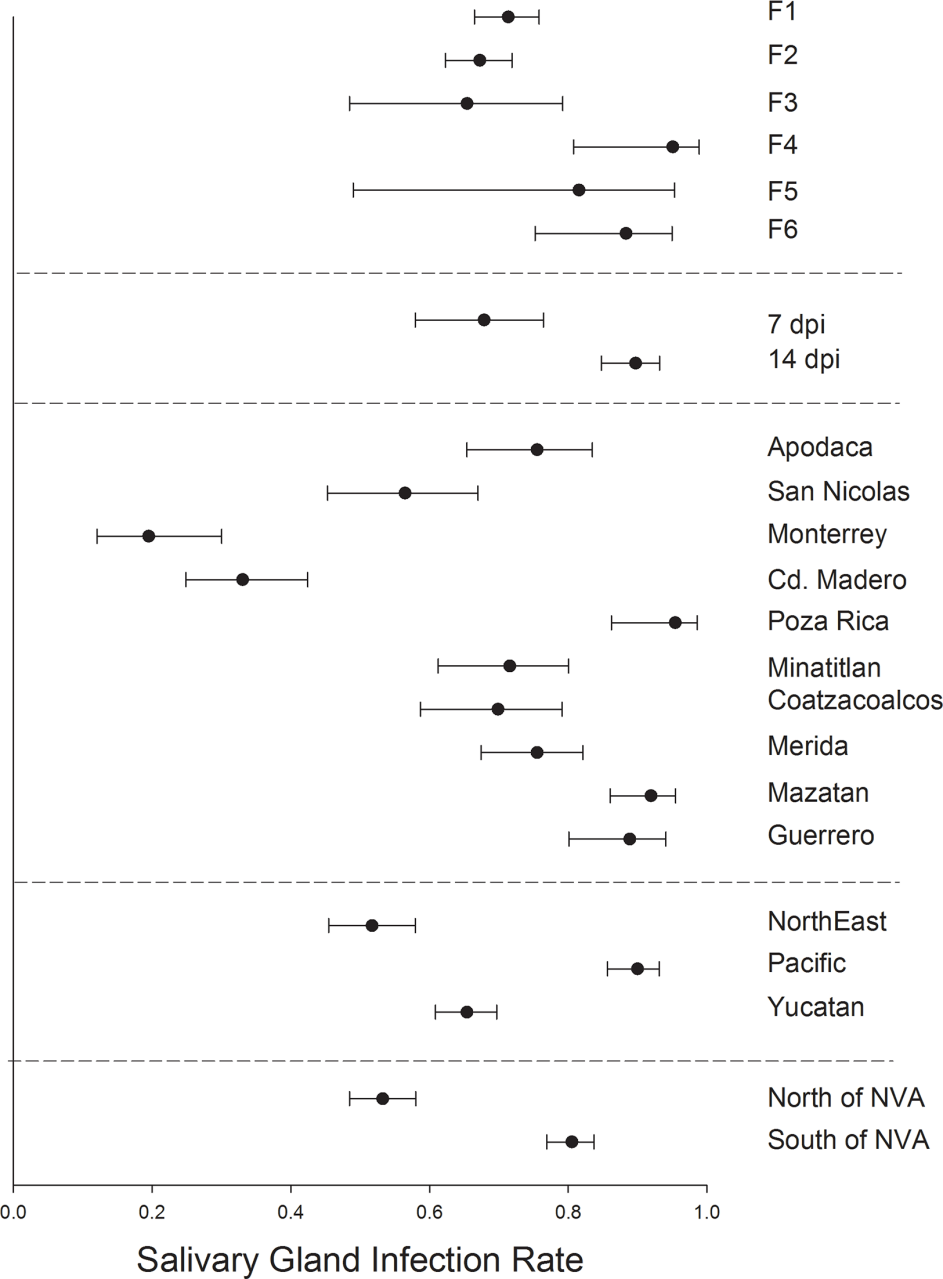
Logistic regression and least square means to compare salivary gland infection rates. 1) among six generations of *Ae*. *aegypti*, 2) between mosquitoes processed at 7 versus 14 dpi, 3) among 10 collections, 4) among 3 regions and 5) between collections north versus south of the NVA.

SGIR varied significantly between mosquito generations F_2_ and F_6_ but otherwise broadly overlapped among the six generations (Fig 5). Mosquitoes harvested at 7 dpi had a lower SGIR than those harvested at 14 dpi. SGIR varied broadly between collections with Ciudad Madero and Monterrey having the lowest SGIR and Poza Rica, Mazatan and Guerrero having the greatest SGIR. SGIR was least in the northeastern region, intermediate in the Yucatan and highest in Pacific collections. SGIR was lowest in collections north of the NVA.

**Fig 6:**
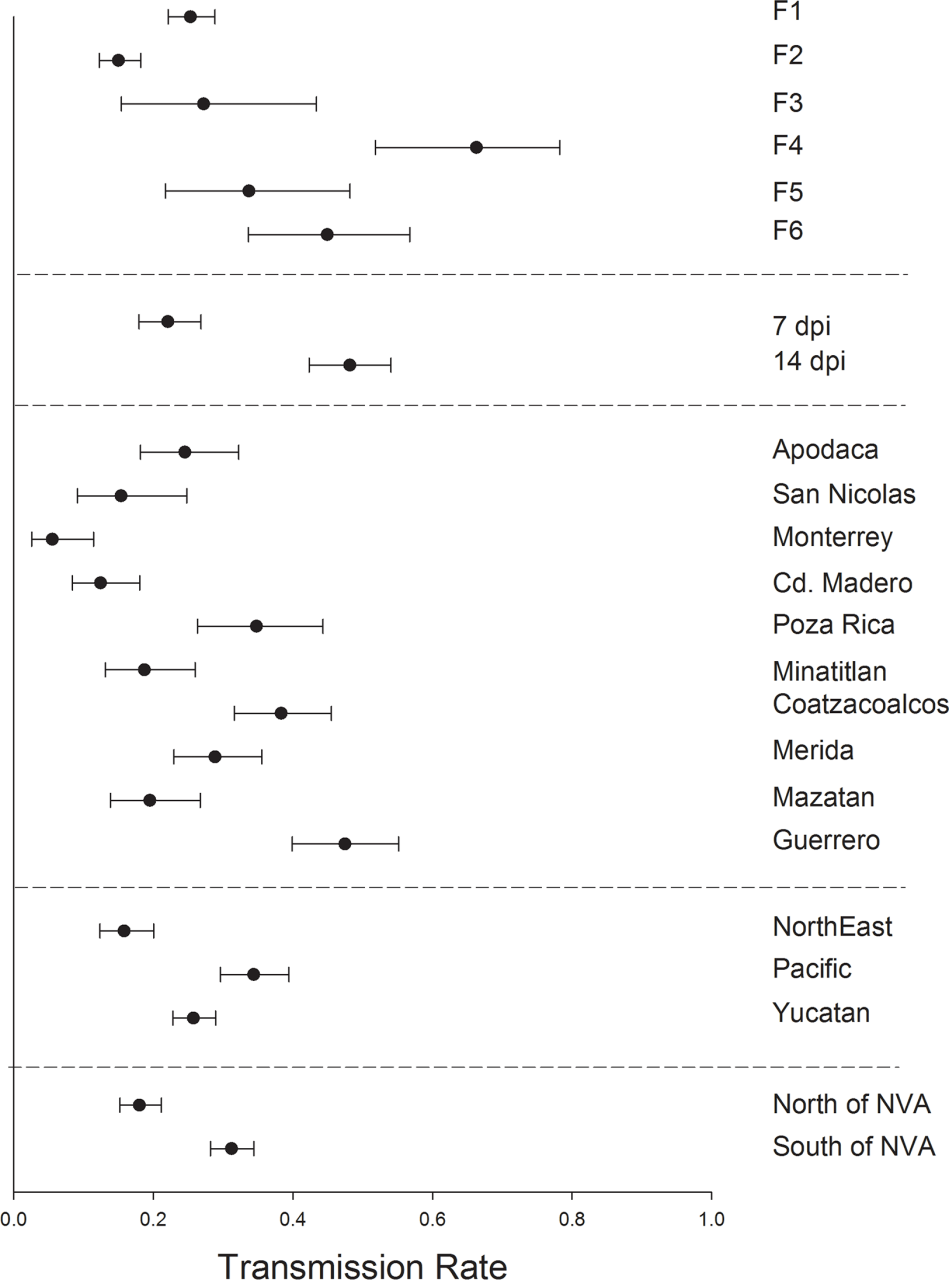
Logistic regression and least square means to compare transmission rates. 1) among six generations of *Ae*. *aegypti*, 2) between mosquitoes processed at 7 versus 14 dpi, 3) among 10 collections, 4) among 3 regions and 5) between collections north versus south of the NVA.

TR varied significantly and broadly among mosquito generations F_1_—F_6_ (Fig 6). Mosquitoes harvested at 7 dpi had a much lower TR than those harvested at 14 dpi. SGIR varied broadly between collections with Ciudad Madero and Monterrey again having the lowest TR and Poza Rica, Coatzacoalcos and Guerrero having the greatest TR. TR, as with the three other measures, was least in the northeastern region, but the same in the Yucatan and Pacific collections. TR was lowest in collections north of the NVA. These patterns could be confounded by the large variation in TR among mosquito generations.

**Fig 7:**
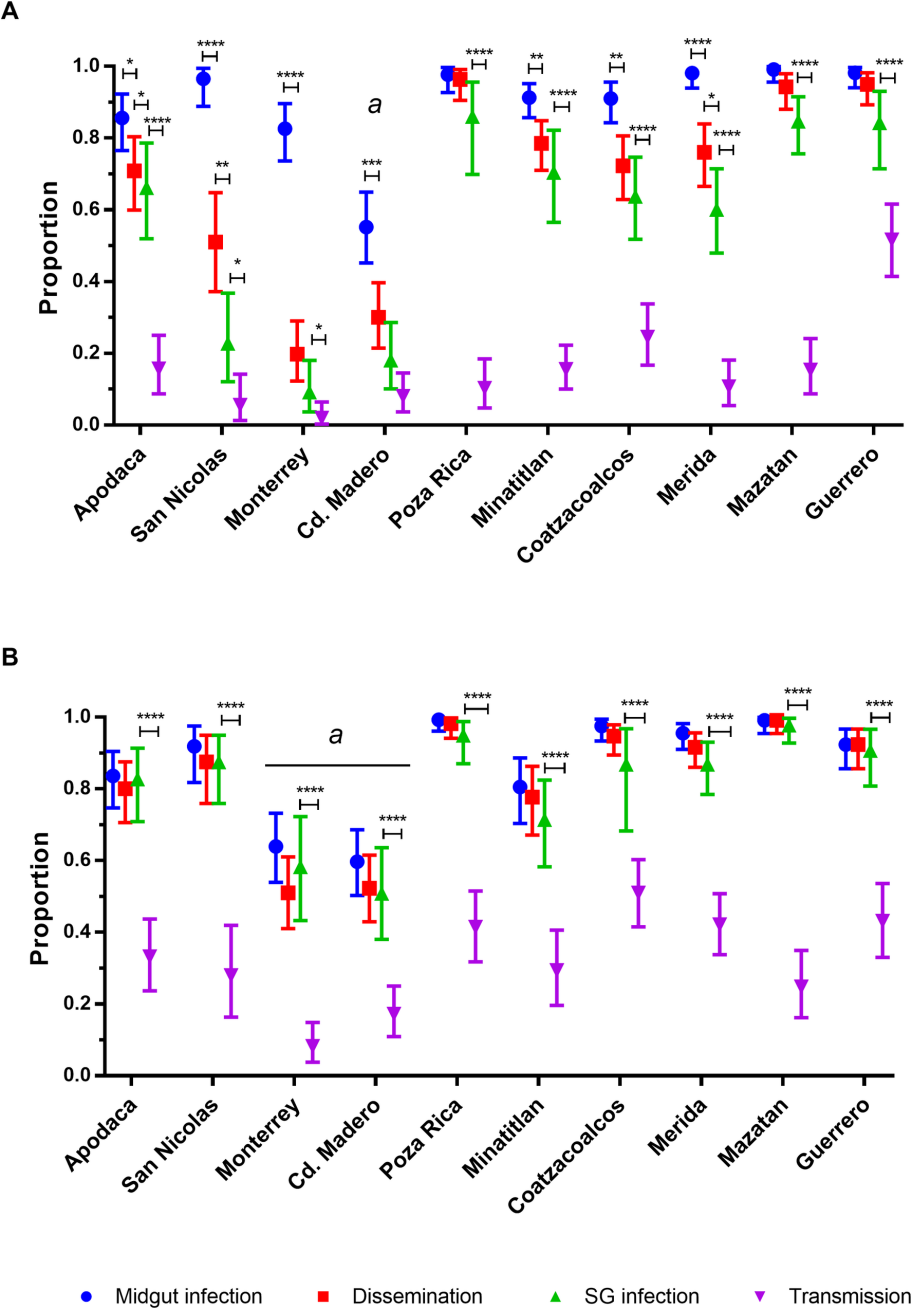
Midgut infection, dissemination, salivary gland infection and transmission rates of *Ae*. *aegypti* by collection. A) rates at 7 dpi and B) 14 dpi by collection location. Apodaca is the furthest northern collection while Guerrero is the furthest south. Error bars represent 95% confidence intervals. Data are from at least 2 independent replicates. Statistical significance is depicted as **** for p <0.0001, *** p<0.001, **p<0.01 and * p<0.05 by two-tailed Fisher’s exact test.

The DIR, MIR, SGIR and TR are shown together for each collection at 7 dpi (Fig 7A) and 14 dpi (Fig 7B). At 7 dpi the MIR was high except for Ciudad Madero. In most cases, the DIR and SGIR were lower than the MIR. This suggests that at 7 dpi most infections have not disseminated yet and only a few DI have progressed to infect the salivary glands. At 14 dpi the MIR, DIR, and SGIR are equivalent except for Ciudad Madero and Monterrey. The TR at 14 dpi are uniformly greater than they were at 7 dpi.

**Fig 8:**
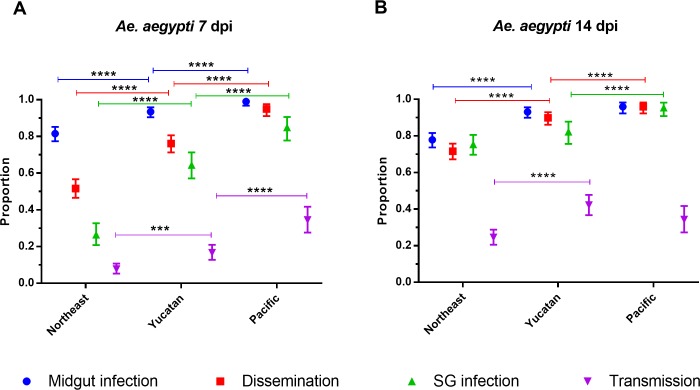
Midgut infection, dissemination, salivary gland infection and transmission rates of *Ae*. *aegypti* by region. A) rates at 7 dpi and B) 14 dpi. Error bars represent 95% confidence intervals. Statistical significance is depicted as **** for p <0.0001, *** p<0.001, **p<0.01 and * p<0.05 by two-tailed Fisher’s exact test.

The DIR, MIR, SGIR and TR are shown together for each region at 7 dpi (Fig 8A) and 14 dpi (Fig 8B). At 7 dpi MIR in Pacific collections are significantly greater than in Yucatan collections which are significantly greater than in northeastern collections. That same pattern occurs in DIR, SGIR, and TR. By 14 dpi the MIR is the same in Pacific and Yucatan collections and both are greater than in northeastern collections. The DIR is the greatest in Pacific collections, significantly higher than in Yucatan collections and least in northeastern collections. The SGIR is the same in Yucatan and northeastern collections; both lower than in the Pacific. The TR in the Pacific and Yucatan collections are the same and both are greater than the TR in northeastern collections.

**Fig 9:**
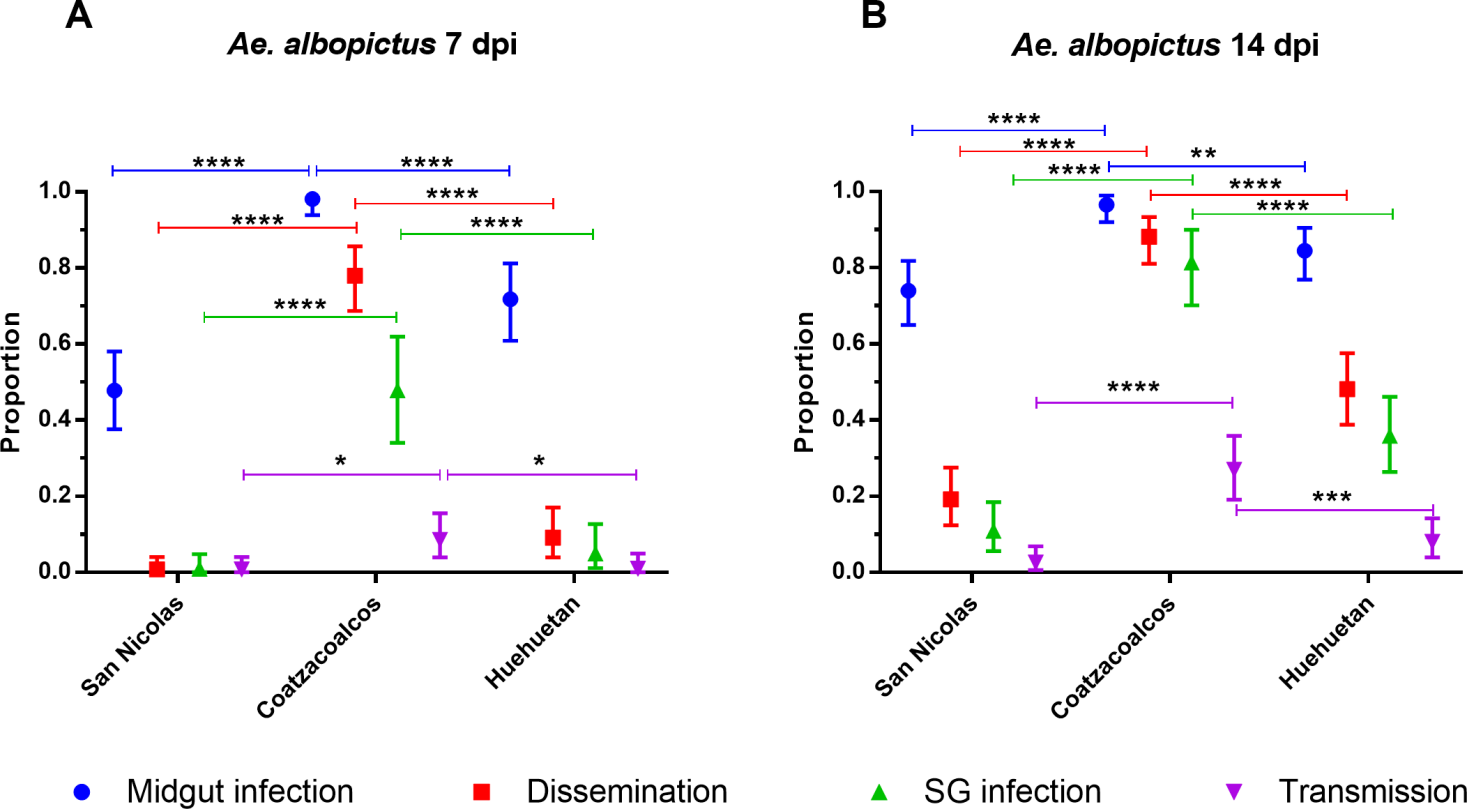
Midgut infection, dissemination, salivary gland infection and transmission rates of *Ae*. *aegypti* by collection. A) rates at 7 dpi and B) 14 dpi by collection location. San Nicolas is the furthest northern collection while Huehuetan is the furthest south. Error bars represent 95% confidence intervals. Data are from at least 2 independent replicates. Statistical significance is depicted as **** for p <0.0001, *** p<0.001, **p<0.01 and * p<0.05 by two-tailed Fisher’s exact test.

**Fig 10:**
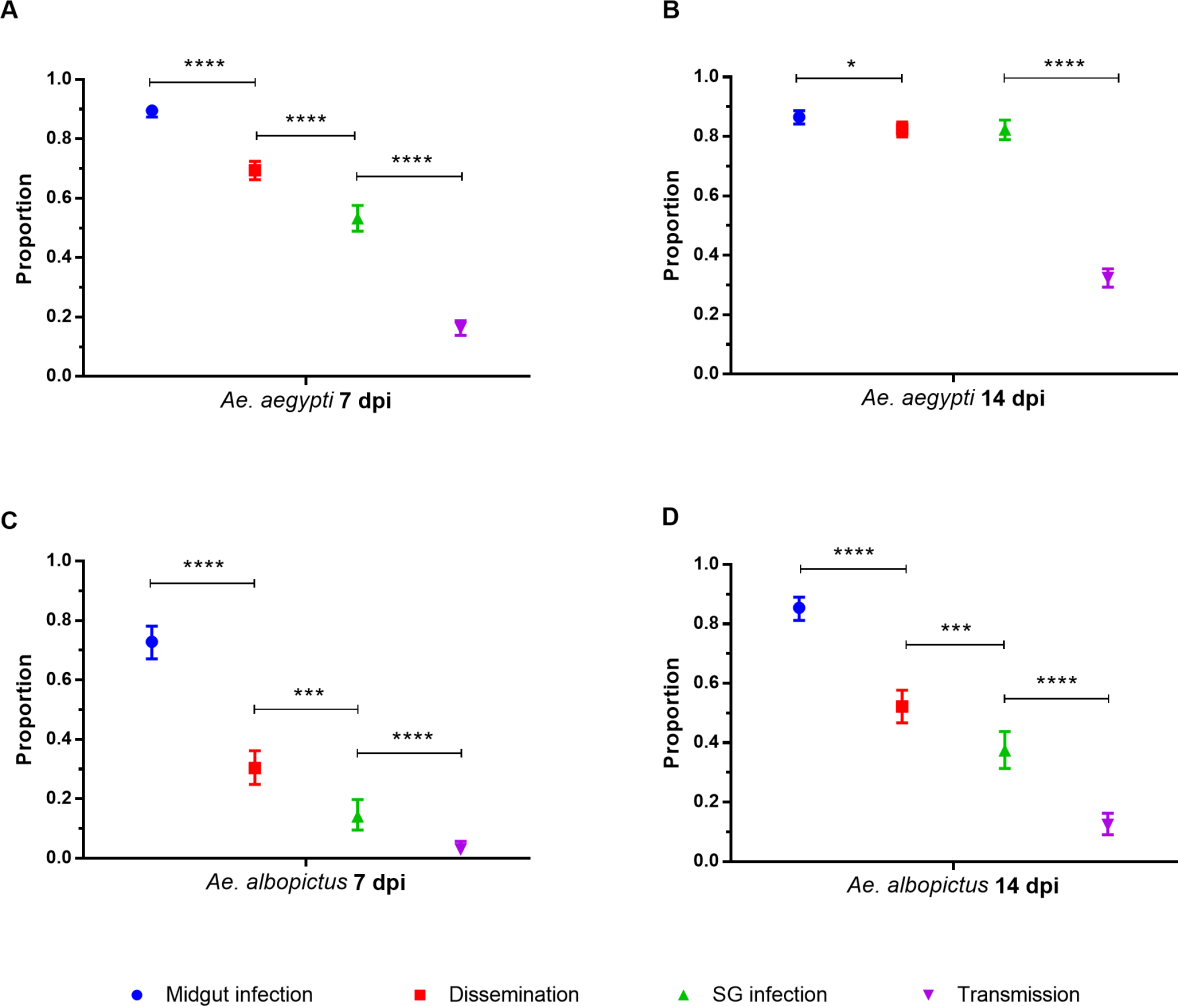
**Midgut infection, dissemination, salivary gland infection and transmission rates among all *Ae*. *aegypti* and *Ae*. *albopictus* collections at 7 and 14 dpi (A and B).** Error bars represent 95% HDI confidence intervals. Statistical significance is depicted as **** for p <0.0001, *** p<0.001, **p<0.01 and * p<0.05 by two-tailed Fisher’s exact test.

Fig 9 displays DIR, MIR, SGIR and TR for each of the three *Ae*. *albopictus* collections at 7 dpi (Fig 9A) and 14 dpi (Fig 9B). The four VC rates were not compared among the three *Ae*. *albopictus* collections because they are confounded by large variation among the generation measured and because the four rates were highly correlated with the log_10_(x+1) blood titer. However it was instructive to compare VC patterns between the two species. At 14 dpi in *Ae*. *aegypti* the MIR, DIR, and SGIR were equivalent (except for Ciudad Madero and Monterrey). However in *Ae*. *albopictus* the MIR, DIR, and SGIR are significantly different at 14d pi in all three collections. This may suggest that in *Ae*. *albopictus* there are distinct MIB, DIB and SGIB that block passage of ZIKV from one tissue to the next. In addition notice that, unlike *Ae*. *aegypti*, all four parameters only increase slightly from 7 dpi to 14dpi. These patterns are summarized for both species in Fig 10 across all collection sites combined to reiterate species differences.

Table 1 shows the relative contribution of each of the four barriers in all 10 collections using Eqs 1–10. Based on the contribution of each barrier, this analysis shows that the main barrier to ZIKV transmission in *Ae*. *aegypti* is the SGEB (Table 1) in nine of the 10 collections and SGIB was the main barrier in Ciudad Madero. TR ranged from 8–51% at 14 dpi. The main barrier to ZIKV transmission in *Ae*. *albopictus* was the SGEB in Coatzacoalcos (Table 1) and SGIBs in Ciudad Nicolas and Huehuetan. TR ranged from 2–21% at 14 dpi in *Ae*. *albopictus*.

**Table 1:** Contribution of each of the barriers (MIB, MEB, SGIB and SGEB) to the overall TR. The contribution of each of the barriers to the overall VC was calculated accordingly to Eqs 1–4. In each row the largest percentages are shown in bold.

Collection	TR	MIB	MEB	SGIB	SGEB
***Aedes aegypti***					
Apodaca	33%	16%	4%	3%	**78%**
San Nicolas	27%	5%	4%	8%	**82%**
Monterrey	8%	17%	9%	20%	**54%**
Cd. Madero	17%	29%	7%	**39%**	25%
Poza Rica	41%	0%	1%	4%	**95%**
Minatitlan	29%	17%	3%	26%	**54%**
Coatzacoalcos	51%	3%	4%	17%	**76%**
Merida	42%	5%	5%	15%	**76%**
Mazatan	24%	0%	0%	1%	**99%**
Guerrero	43%	9%	0%	11%	**81%**
***Aedes albopictus***					
San Nicolas	2%	8%	35%	**57%**	0%
Coatzacoalcos	27%	2%	7%	15%	**76%**
Huehuetan	8%	6%	22%	**40%**	31%

## Discussion

This study demonstrates that Mexican *Ae*. *aegypti* and *Ae*. *albopictus* are competent vectors of ZIKV. However the patterns of MIR, DIR, SGIR and TR in general vary between the two species. The MIR, DIR, and SGIR were all statistically similar by 14 dpi in *Ae*. *aegypti* (Fig 10). In contrast in *Ae*. *albopictus*, the MIR, DIR, and SGIR are significantly different at 14dpi in all three collections. In *Ae*. *albopictus* there may be distinct MIB, DIB and SGIB that block passage of ZIKV from one tissue to the next. In addition notice that, unlike *Ae*. *aegypti*, all four rates only increase slightly from 7 dpi to 14dpi in *Ae*. *albopictus*.

Vector competence varied among collections and geographic regions in Mexico and depending on whether mosquitoes are collected north or south of the NVA. Lozano-Fuentes et al [26] previously reported an abrupt decline in MIR, DIR, SGIR and TR in collections just south of the NVA. This was consistent with a hypothesis that the intersection of the NVA with the Gulf of Mexico coast acts as a barrier to gene flow previously observed between *Ae*. *aegypti* collections north and south on coastal plain along the Gulf of Mexico. The Transverse Volcanic Belt of Mexico divides the state of Veracruz into northern and southern Coastal Plains. This belt began to develop during the Oligocene and then later, during the Pliocene–Pleistocene, intense orogenic activity raised the Neovolcanic axis. The NVA extends from near the Pacific Coast east to the Gulf of Mexico and intersects the Atlantic coast. In the present study an abrupt drop in MIR, DIR and SGIR was observed but far north of the NVA in Monterrey and Ciudad Madero. On the other hand, the lower VC in northeastern collections observed by Bennett et al [31] for DENV2 were also noted in the current paper for ZIKV.

In general both *Ae*. *aegypti* and *Ae*. *albopictus* were highly susceptible to midgut infection, had high dissemination and salivary gland infection rates but these led to only low to moderate transmission rates. The potential role of the SG barriers has been previously suggested for ZIKV [23, 32], and herein we also provide evidence of a strong SGEB limiting ZIKV transmission in Mexican *Ae*. *aegypti* populations. Salivary gland escape barriers may therefore be the most important factors limiting ZIKV transmission by *Ae*. *aegypti*. In contrast *Ae*. *albopictus* had lower rates of midgut infection and dissemination but transmission was limited mainly by a salivary gland infection barrier.

TR at 14 dpi varied from 8–51% in *Ae*. *aegypti* and from 2–26% in *Ae*. *albopictus*. SGEBs are indicated when mosquitoes have an infected salivary gland but are unable to transmit virus. The existence of SGEBs has been definitively demonstrated for Japanese Encephalitis Virus in *Culex tritaeniorhynchus*[33], Snow Shoe Hare virus in *Ae*. *triseriatus*[34], La Crosse virus (LACV) in *Ae*. *hendersoni* [35] and Sindbis virus in *Cx*.*theileri* [36]. More recently, SGEBs have been reported for Rift Valley Fever Virus [37, 38].

Alternatively, an apparent SGEB may simply reflect a low sensitivity of saliva collection for detecting live ZIKV followed by plaque assays. A major difference between studies of SGEB made in the mid 1970–1980’s and similar studies done now is that earlier studies were able to use 8-day-old (suckling) mice to test for arboviral transmission since suckling mice are susceptible to fatal infection. Although there are recent examples [39], use of suckling mice is now prohibited by many institutions including CSU for bioethical reasons. Use of suckling mice is also prohibitively expensive when analyzing many mosquitoes as in the current study.

The protocol used in the present study involves removing the legs and wings of the mosquito and sliding its proboscis into the narrow end of a glass capillary tube filled with mineral oil. This “oil-technique” is well established at CSU and can be viewed as an online video which shows CSU personnel performing the technique [40]. Plaque assays are then used to detect live virus and/or measure the amount of live virus in the collected saliva. However this quantitative step was not taken in the present study because of the large numbers of individuals analyzed.

Measuring TR in collected saliva remains problematic because mosquitoes vary in the amount of saliva that they produce during probing and ingestion of a blood meal. Furthermore the concentration of virus may not be uniform in the saliva such that small volumes may carry as much virus as a larger volume. As examples, transmission electron microscopy in the salivary glands of *Ae*. *albopictus* revealed mature CHIKV particles (an alphavirus) that are highly clumped (almost crystalized) in some acinar cells [41] but in other cells mature particles appear to be more uniformly distributed. The same pattern appears in the salivary glands of *Culex pipiens quinquefasciatus* [42] infected with West Nile Virus (a flavivirus).

However, using transmission electron microscopy can also be misleading because many of the particles that are visualized may be defective rather than live virus. Defective particles are also a problem when trying to quantify viral RNA using Quantitative RT-PCR [43]. With DENV-2, we found that lower detection and quantitation limits were 20 and 200 copies per reaction, respectively. Amounts of positive and negative strand viral RNA strands were correlated and the numbers of plaque-forming units (PFU) were correlated with DENV-2 RNA copy number in both C6/36 cell cultures and mosquitoes. PFU were consistently lower than RNA copy number by 2–3 log_10_. Some investigators use pilocarpine (a parasympathomimetic plant alkaloid) to promote salivation [44] when attempting to collect saliva. But in our experience, while pilocarpine increases the volume of saliva produced by a mosquito, it also largely depletes the salivary glands of saliva and makes them brittle and difficult to remove intact. Furthermore, pilocarpine treated females probably produce more saliva than they probably do in a normal bite. Thus it is possible that the salivary escape barriers reported here are due to lack of sensitivity of our in-vitro assay for virus transmission.

It would be interesting to repeat this study using ZIKV isolates from Mexico. At the time that we initiated this study no viral isolates from Mexico were available. Genotype x genotype interactions have been observed for other flaviviruses [45]. However, it has been also observed that virus from the Asian strain behaves similarly to a virus from the Americas (Mexico) [24]. A strain from Mexico and the Puerto Rican PRVABC59 was previously shown to be very similar [29].

## Supporting information

S1 Table Raw data. sorted according to sampling location, species, the number of generations the mosquito had spent in the laboratory, days post infection, titer of virus in the blood-meal and the number of infected midguts, legs, salivary glands and saliva. N is the sample size which is > N salivary glands because the salivary gland was occasionally lost or destroyed during dissection.
(DOCX)

S2 Table Primer and probe sequences for the ZIKV 3’ UTR assay. (DOCX)

S1 Fig Logistic regression and least square means to compare midgut infection rates. 1) among three generations of *Ae*. *albopictus*, 2) between mosquitoes processed at 7 versus 14 dpi, 3) among three collections, 4) among 3 regions and 5) between collections north versus south of the NVA.
(TIF)

S2 Fig Logistic regression and least square means to compare disseminated infection rates. 1) among three generations of *Ae*. *albopictus*, 2) between mosquitoes processed at 7 versus 14 dpi, 3) among three collections, 4) among 3 regions and 5) between collections north versus south of the NVA.
(TIF)

S3 Fig Logistic regression and least square means to compare salivary gland infection rates. 1) among three generations of *Ae*. *albopictus*, 2) between mosquitoes processed at 7 versus 14 dpi, 3) among three collections, 4) among 3 regions and 5) between collections north versus south of the NVA.
(TIF)

S4 Fig Logistic regression and least square means to compare transmission rates. 1) among three generations of *Ae*. *albopictus*, 2) between mosquitoes processed at 7 versus 14 dpi, 3) among three collections, 4) among 3 regions and 5) between collections north versus south of the NVA.
(TIF)
