# Supplementary material for: Variation in competence for ZIKV transmission by Aedes aegypti and Aedes albopictus in Mexico
Source: PLoS Negl Trop Dis. 2018 Jul 2;12(7):e0006599. doi: 10.1371/journal.pntd.0006599 (PMC6044546; doi:10.1371/journal.pntd.0006599)
Supplement: S1 Table — sorted according to sampling location, species, the number of generations the mosquito had spent in the laboratory, days post infection, titer of virus in the blood-meal and the number of infected midguts, legs, salivary glands and saliva. N is the sample size which is > N salivary glands because the salivary gland was occasionally lost or destroyed during dissection. (DOCX) [file pntd.0006599.s001.docx]

| Location | Specie | Generation | dpi | Blood titer | Positive  midguts | Positive  legs | Positive  saliva | N | Positive salivary glands | N  Salivary glands |
| --- | --- | --- | --- | --- | --- | --- | --- | --- | --- | --- |
| Apodaca | Aae | F1 | 7 | 1.50E+06 | 25 | 24 | 5 | 25 | 16 | 19 |
| Apodaca | Aae | F2 | 7 | 1.80E+07 | 38 | 28 | 6 | 48 | 14 | 26 |
| Apodaca | Aae | F1 | 14 | 1.50E+06 | 34 | 34 | 16 | 34 | 24 | 24 |
| Apodaca | Aae | F2 | 14 | 1.80E+07 | 35 | 32 | 11 | 48 | 17 | 25 |
| San Nicolas | Aae | F2 | 7 | 4.00E+05 | 24 | 8 | 1 | 24 | 2 | 20 |
| San Nicolas | Aae | F3 | 7 | 2.00E+07 | 22 | 16 | 1 | 23 | 7 | 21 |
| San Nicolas | Aae | F2 | 14 | 4.00E+05 | 21 | 20 | 1 | 24 | 20 | 24 |
| San Nicolas | Aae | F3 | 14 | 2.00E+07 | 20 | 19 | 11 | 20 | 19 | 20 |
| San Nicolas | Aal | F3 | 7 | 8.80E+05 | 22 | 0 | 0 | 48 | 0 | 39 |
| San Nicolas | Aal | F4 | 7 | 8.00E+05 | 20 | 0 | 0 | 40 | 0 | 35 |
| San Nicolas | Aal | F3 | 14 | 8.80E+05 | 38 | 12 | 1 | 41 | 7 | 31 |
| San Nicolas | Aal | F4 | 14 | 8.00E+05 | 37 | 7 | 1 | 60 | 2 | 56 |
| Monterrey | Aae | F2 | 7 | 4.40E+07 | 29 | 4 | 0 | 39 | 1 | 34 |
| Monterrey | Aae | F2 | 7 | 8.00E+05 | 40 | 12 | 1 | 44 | 4 | 26 |
| Monterrey | Aae | F2 | 14 | 8.00E+05 | 28 | 20 | 6 | 44 | 11 | 22 |
| Cd. Madero | Aae | F1 | 7 | 6.20E+05 | 32 | 17 | 6 | 48 | 7 | 30 |
| Cd. Madero | Aae | F1 | 7 | 8.00E+05 | 20 | 11 | 1 | 46 | 4 | 33 |
| Cd. Madero | Aae | F1 | 14 | 6.20E+05 | 41 | 39 | 15 | 60 | 19 | 29 |
| Cd. Madero | Aae | F1 | 14 | 8.00E+05 | 23 | 17 | 3 | 47 | 9 | 26 |
| Poza Rica | Aae | F1 | 7 | 1.80E+07 | 48 | 47 | 5 | 48 | 22 | 25 |
| Poza Rica | Aae | F1 | 14 | 1.40E+05 | 19 | 19 | 2 | 19 | 14 | 14 |
| Poza Rica | Aae | F1 | 14 | 1.80E+07 | 48 | 48 | 33 | 48 | 27 | 27 |
| Minatitlan | Aae | F1 | 7 | 1.60E+06 | 38 | 35 | 7 | 39 | 23 | 24 |
| Minatitlan | Aae | F1 | 7 | 6.20E+05 | 40 | 27 | 2 | 47 | 9 | 21 |
| Minatitlan | Aae | F1 | 14 | 1.60E+06 | 18 | 18 | 5 | 21 | 15 | 18 |
| Minatitlan | Aae | F1 | 14 | 6.20E+05 | 38 | 36 | 15 | 48 | 21 | 32 |
| Coatzacoalcos | Aae | F1 | 7 | 1.40E+05 | 48 | 43 | 19 | 50 | 30 | 41 |
| Coatzacoalcos | Aae | F2 | 7 | 1.70E+06 | 39 | 26 | 4 | 45 | 11 | 23 |
| Coatzacoalcos | Aae | F1 | 14 | 1.40E+05 | 75 | 73 | 36 | 77 | 17 | 19 |
| Coatzacoalcos | Aae | F1 | 14 | 1.70E+06 | 29 | 28 | 18 | 29 | 0 | 0 |
| Coatzacoalcos | Aal | F2 | 7 | 1.50E+06 | 40 | 28 | 2 | 41 | 7 | 23 |
| Coatzacoalcos | Aal | F2 | 7 | 2.00E+06 | 47 | 41 | 5 | 47 | 15 | 23 |
| Coatzacoalcos | Aal | F2 | 14 | 1.50E+06 | 43 | 42 | 15 | 45 | 19 | 20 |
| Coatzacoalcos | Aal | F2 | 14 | 2.00E+06 | 36 | 34 | 7 | 36 | 17 | 18 |
| Coatzacoalcos | Aal | F3 | 14 | 8.80E+05 | 23 | 17 | 6 | 24 | 10 | 18 |
| Merida | Aae | F1 | 7 | 1.80E+07 | 44 | 37 | 8 | 45 | 23 | 32 |
| Merida | Aae | F2 | 7 | 4.40E+07 | 44 | 31 | 1 | 44 | 15 | 31 |
| Merida | Aae | F1 | 14 | 1.80E+07 | 29 | 29 | 16 | 30 | 16 | 17 |
| Merida | Aae | F2 | 14 | 4.40E+07 | 48 | 45 | 20 | 48 | 28 | 32 |
| Merida | Aae | F2 | 14 | 8.00E+05 | 44 | 42 | 17 | 48 | 26 | 31 |
| Mazatan | Aae | F2 | 7 | 1.12E+07 | 48 | 46 | 8 | 48 | 41 | 44 |
| Mazatan | Aae | F2 | 7 | 4.40E+07 | 33 | 31 | 4 | 33 | 23 | 31 |
| Mazatan | Aae | F2 | 14 | 1.12E+07 | 46 | 46 | 8 | 46 | 42 | 42 |
| Mazatan | Aae | F2 | 14 | 4.40E+07 | 32 | 32 | 11 | 32 | 31 | 32 |
| Huehuetan | Aal | F2 | 7 | 8.80E+05 | 24 | 4 | 0 | 24 | 2 | 20 |
| Huehuetan | Aal | F3 | 7 | 8.00E+05 | 28 | 2 | 0 | 48 | 0 | 33 |
| Huehuetan | Aal | F2 | 14 | 8.80E+05 | 43 | 28 | 6 | 48 | 18 | 39 |
| Huehuetan | Aal | F3 | 14 | 8.00E+05 | 47 | 23 | 2 | 58 | 13 | 48 |
| Guerrero | Aae | F4 | 7 | 2.00E+06 | 47 | 46 | 25 | 48 | 19 | 21 |
| Guerrero | Aae | F6 | 7 | 1.80E+07 | 43 | 41 | 22 | 43 | 16 | 20 |
| Guerrero | Aae | F5 | 14 | 2.00E+06 | 44 | 44 | 23 | 48 | 18 | 20 |
| Guerrero | Aae | F6 | 14 | 1.80E+07 | 36 | 36 | 14 | 38 | 27 | 29 |
|  |  |  |  |  |  |  |  |  |  |  |
