# Supplementary material for: Variation in competence for ZIKV transmission by Aedes aegypti and Aedes albopictus in Mexico
Source: PLoS Negl Trop Dis. 2018 Jul 2;12(7):e0006599. doi: 10.1371/journal.pntd.0006599 (PMC6044546; doi:10.1371/journal.pntd.0006599)
Supplement: S2 Table — (DOCX) [file pntd.0006599.s002.docx]

Supplementary Table 2. Primer and probe sequences for the ZIKV 3’ UTR assay

|  | **Sequence (5'**🡪**3')** | **Length** | **Start** | **Stop** | **TM (°C)** |
| --- | --- | --- | --- | --- | --- |
| **Forward primer** | CCCAGGAGAAGCTGGGAAAC | 20 | 10453 | 10472 | 60 |
| **Reverse primer** | TCGCCACCTTCTTTTCCCAT | 20 | 10581 | 10600 | 60 |
| **Probe_10,514** | GCCATGCTGCCTGTGAGCCCCT | 19 | 10514 | 10535 | 69.7 |
