# Supplementary figures and images for: Variation in competence for ZIKV transmission by Aedes aegypti and Aedes albopictus in Mexico
Source: PLoS Negl Trop Dis. 2018 Jul 2;12(7):e0006599. doi: 10.1371/journal.pntd.0006599 (PMC6044546; doi:10.1371/journal.pntd.0006599)

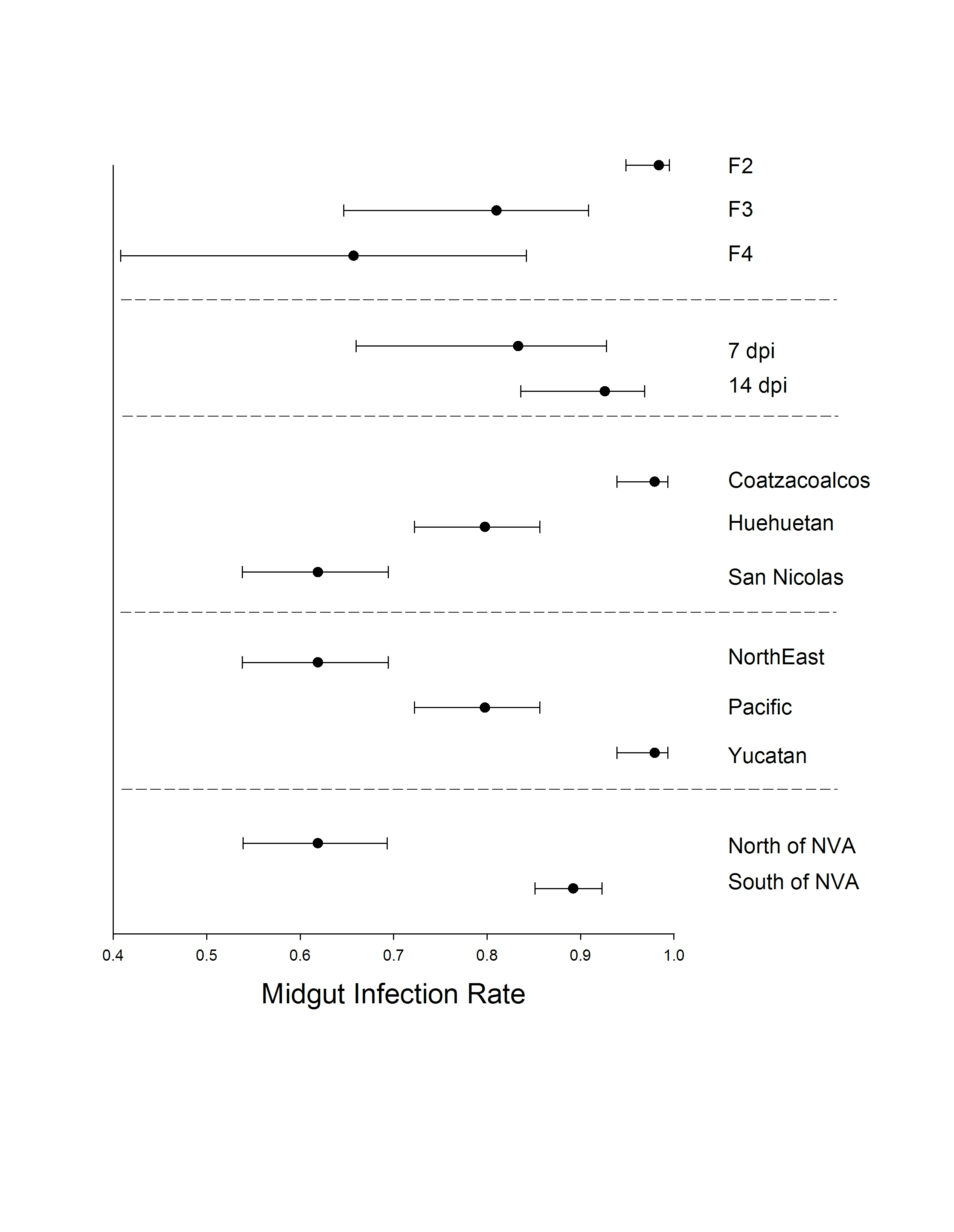

Supplement: S1 Fig — 1) among three generations of Ae. albopictus, 2) between mosquitoes processed at 7 versus 14 dpi, 3) among three collections, 4) among 3 regions and 5) between collections north versus south of the NVA. (TIF) [file pntd.0006599.s003.TIF]

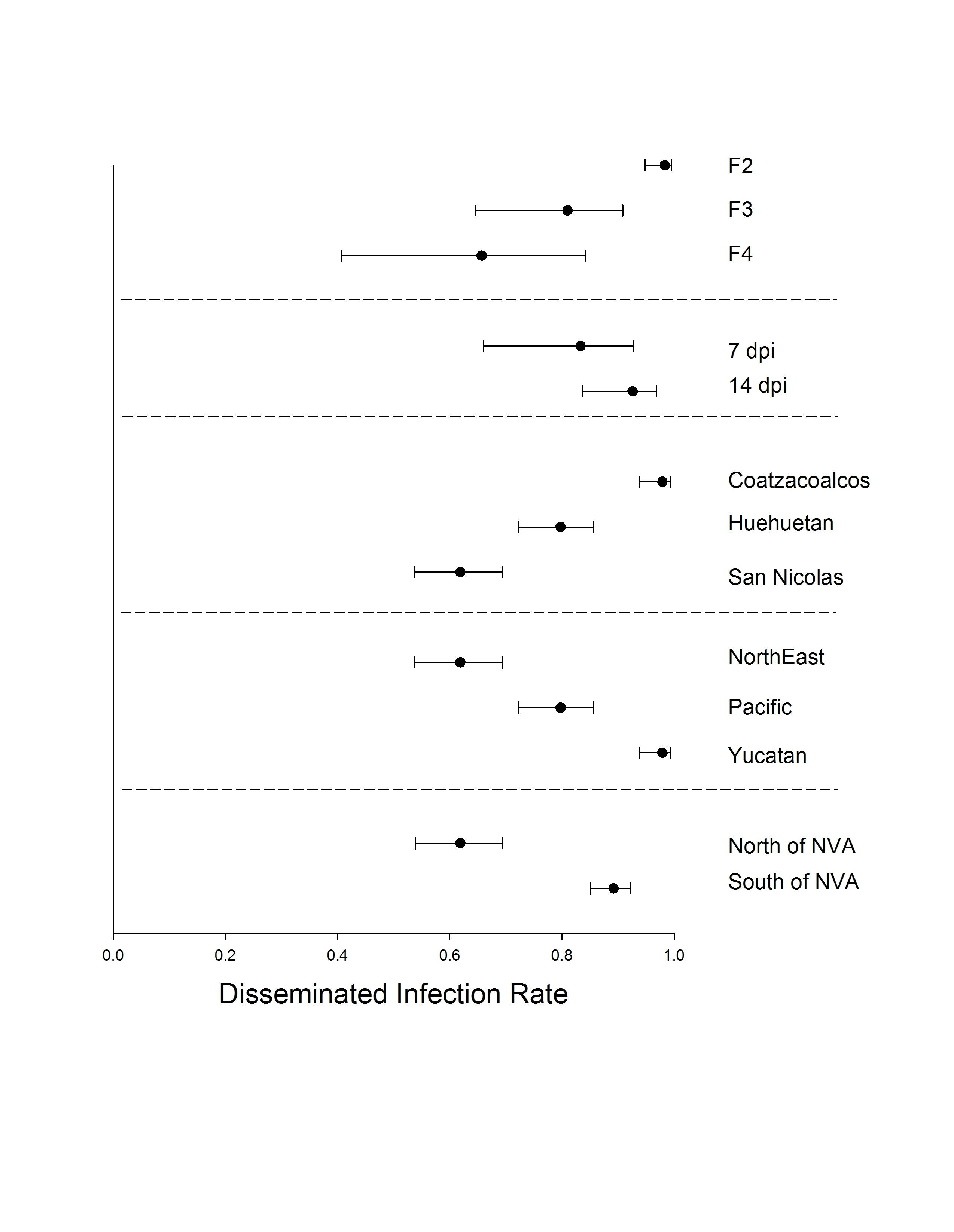

Supplement: S2 Fig — 1) among three generations of Ae. albopictus, 2) between mosquitoes processed at 7 versus 14 dpi, 3) among three collections, 4) among 3 regions and 5) between collections north versus south of the NVA. (TIF) [file pntd.0006599.s004.TIF]

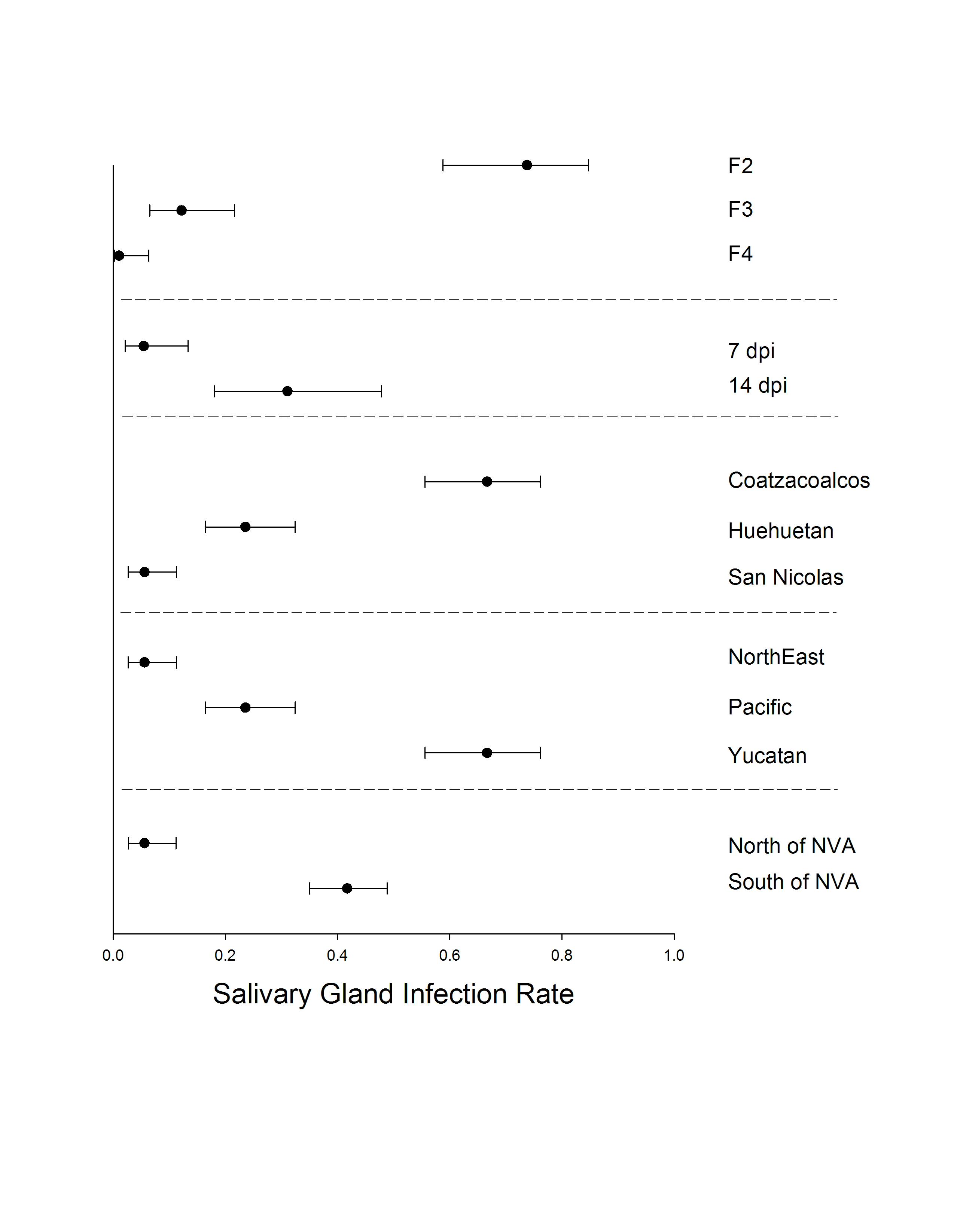

Supplement: S3 Fig — 1) among three generations of Ae. albopictus, 2) between mosquitoes processed at 7 versus 14 dpi, 3) among three collections, 4) among 3 regions and 5) between collections north versus south of the NVA. (TIF) [file pntd.0006599.s005.TIF]

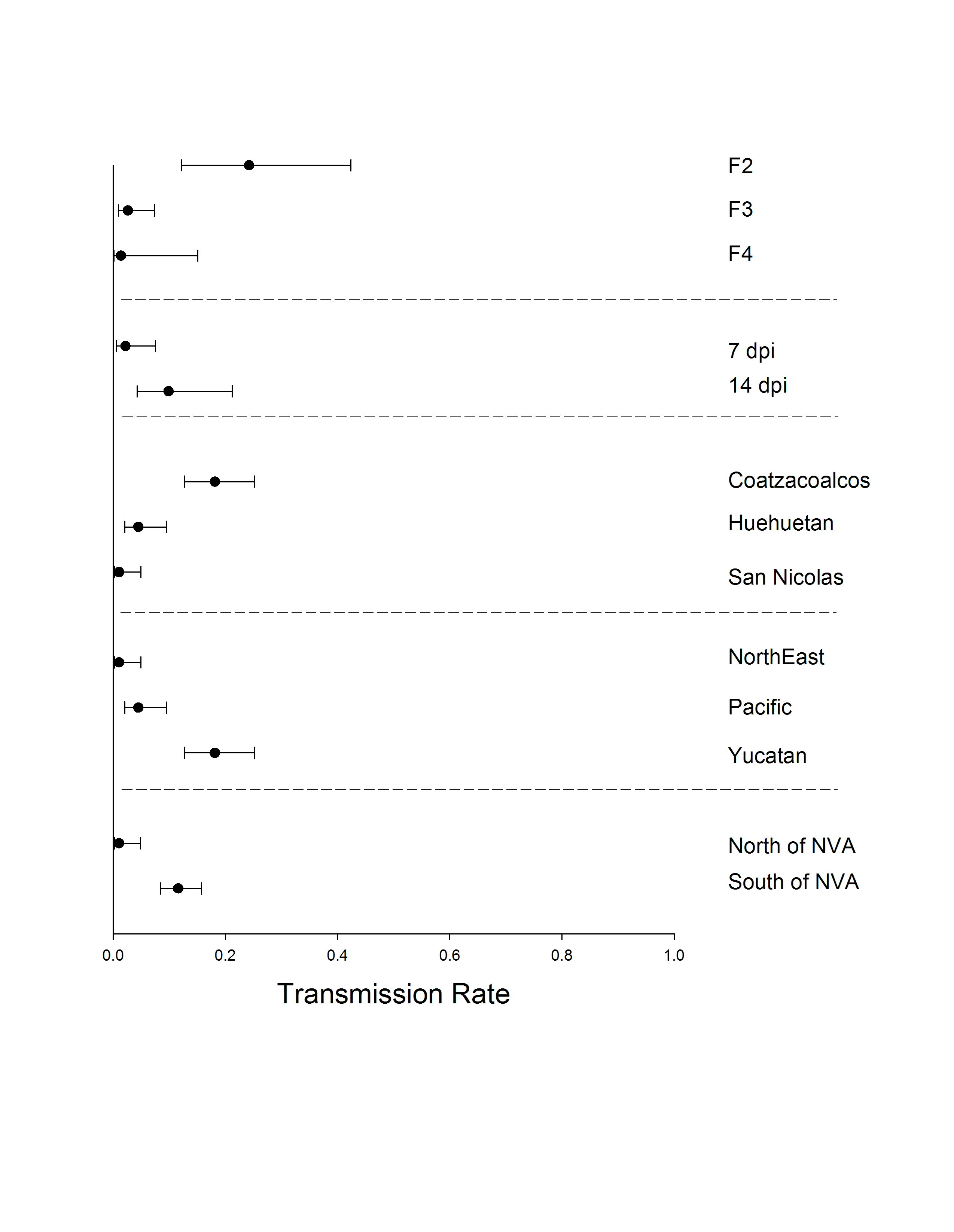

Supplement: S4 Fig — 1) among three generations of Ae. albopictus, 2) between mosquitoes processed at 7 versus 14 dpi, 3) among three collections, 4) among 3 regions and 5) between collections north versus south of the NVA. (TIF) [file pntd.0006599.s006.TIF]
